# Is Housing a Health Insult?

**DOI:** 10.3390/ijerph14060567

**Published:** 2017-05-26

**Authors:** Emma Baker, Andrew Beer, Laurence Lester, David Pevalin, Christine Whitehead, Rebecca Bentley

**Affiliations:** 1 School of Architecture and Built Environment, University of Adelaide, Adelaide 5005, Australia; Laurence.lester@adelaide.edu.au; 2 University of South Australia Business School, Adelaide 5000, Australia; Andrew.beer@unisa.edu.au; 3 School of Health and Human Sciences, University of Essex, Colchester CO4 3SQ, UK; pevalin@essex.ac.uk; 4 Department of Economics, London School of Economics and Political Science, London WC2A 2AE, UK; C.M.E.Whitehead@lse.ac.uk; 5 Melbourne School of Population and Global Health, University of Melbourne, Melbourne 3010, Australia; brj@unimelb.edu.au

**Keywords:** housing, health, index, longitudinal

## Abstract

In seeking to understand the relationship between housing and health, research attention is often focussed on separate components of people’s whole housing ‘bundles’. We propose in this paper that such conceptual and methodological abstraction of elements of the housing and health relationship limits our ability to understand the scale of the accumulated effect of housing on health and thereby contributes to the under-recognition of adequate housing as a social policy tool and powerful health intervention. In this paper, we propose and describe an index to capture the means by which housing bundles influence health. We conceptualise the index as reflecting accumulated housing “insults to health”—an Index of Housing Insults (IHI). We apply the index to a sample of 1000 low-income households in Australia. The analysis shows a graded association between housing insults and health on all outcome measures. Further, after controlling for possible confounders, the IHI is shown to provide additional predictive power to the explanation of levels of mental health, general health and clinical depression beyond more traditional proxy measures. Overall, this paper reinforces the need to look not just at separate housing components but to embrace a broader understanding of the relationship between housing and health.

## 1. Introduction

Since the foundation work of John Snow [1] almost 200 years ago, our conceptualisation of the means by which housing contributes to the health and wellbeing of people has evolved through the work of geographers, epidemiologists, economists and specialist housing researchers. Snow’s work changed the way disease transmission was understood by looking beyond the individual to the place in which they lived. Two centuries of subsequent research has developed the basic understanding of a link between dwelling and disease transmission towards a more fine-grained conceptualization of the role of housing as a determinant of health and wellbeing [2,3].

Housing is more than just shelter. It is a collection of components that together affect individuals’ lives—across and beyond our wealth, health, wellbeing, employment and educational opportunities. This collection of components has been usefully conceptualised (for example [4,5,6]) as a “housing bundle”, one that captures the housing choices, history, available resources and limitations that individuals command. While acknowledging that individual housing components work together as housing bundles, work in the field has mostly focussed on the effects of singular dimensions of housing, such as housing tenure, or affordability, or housing quality, or neighbourhood characteristics and their influence on particular outcomes for individuals, households or populations. This separation of discrete elements within the “housing bundle” is especially evident in the growing body of research examining the relationship between housing and health. This literature considers various aspects of housing—for example location, structure and condition of the dwelling—and has recast our understanding of the housing and health relationship. Much of this research suggests that these separate aspects of housing have little, if any, significant impact on health at the population level in developed economies with benign climates and generally good quality housing (for example Australia [7]). That said, it is clear that substantial sub-populations within nations are vulnerable to the health effects of housing [8], for example long-term tenants, sole supporting parents, persons with a disability, or Indigenous persons.

The premise of this paper is that the conceptual and methodological abstraction of the housing and health relationship limits our ability to understand the relationship between the two. By separating out and then measuring separate components of people’s housing bundles, we are at risk of under-estimating the scale of the overall impact of poor housing upon health and ignoring important interactions between parts of housing bundles. Critically, we suggest that by focussing investigation on separate components of housing bundles, we may be contributing to the under-recognition of adequate housing as a social policy tool and powerful health intervention.

### 1.1. Housing Bundles vs. Abstracted Components 

This paper contributes to a growing body of work that aims to capture and measure the ways that housing influences our lives, in particular our health and wellbeing. Unanimously, work within this field acknowledges the complexity of the housing and health relationship and the difficulties of isolating and measuring the individual health effects of housing from within the diverse complexity of people’s lives [9].

In beginning to understand and measure the relationship, analyses have predominantly conceptualised and analysed the complex interplay between housing and health through a process we refer to as “abstraction”. In this process, parts of the housing bundle are statistically isolated and measured while controlling for external effects (such as poverty or education). The guiding rationale in such analyses has been to understand the underlying drivers by quantifying each of the components separately. Such analyses have for example shown that there is a measurable mental health effect (with inferred causality) of residing in unaffordable housing [10,11] or that there is a directional relationship between damp dwellings and respiratory health [12,13,14] or that there is an effect attributable to tenure mix on labour market outcomes [15].

The social determinants of health framework [16] has been a powerful means to broaden the understanding of health from a focus only on the direct causes of pathology and disease, towards incorporating the important pathways of behaviour, environment and resources. This movement has highlighted the importance of living and working conditions in influencing health outcomes, and within it housing is considered a “key social determinant of health” (for example [17]). The acknowledgement of housing as a social determinant among social epidemiologists has, to a large extent, methodologically shaped much recent analysis. Researchers within (or influenced by) this field have approached the measurement of the relationship between housing and health from within a social determinants framework (as famously pictorialized by Dahlgren and Whitehead [18]), where health is shaped by age, sex, lifestyle and constitutional factors, social and community networks, socio-economic, environmental, cultural, and living and working conditions. Within this framework, housing is regarded as just one of the eight sub-components of living and working conditions.

Importantly, this body of work is also strongly influenced by the methods employed by epidemiologists, which predominantly seek to build understanding by isolating the effects of individual determinants and measuring specific health outcomes using large samples and statistically robust techniques. While acknowledging the contribution of such approaches to building a new, and often policy relevant, evidence base on the relationship, we suggest this conceptualisation and its inherent methodologies may eventually be unhelpful in understanding and measuring the relationship between housing and health. To a degree, our conceptualisation of the relationship between housing and health has been “medicalised” [19] through a narrow focus on identifying the impacts of single points of interventions, rather than adopting a broader perspective that examines the relationship in toto. In part, these are questions of epistemology, with positivist philosophies of science overshadowing realist [20] and other perspectives on knowledge.

While such frameworks attempt to capture a ‘universal’ understanding of the factors that work in concert to influence health, the identification and separation of the component determinants implies that they can be separated and effects measured independently. The attention given to the influence of individual determinants may also misrepresent the nature of the relationship between housing and health. In focussing only on small, distinct parts of the housing and health relationship, the impacts (effect sizes) measured after controlling for other influences (such as income) are necessarily small (but often highly statistically significant). Though we assume that these small effects accumulate (across a collection of other distinct components of the housing and health relationship) towards much larger effects in individual lives, the case for understanding the combined impact of housing on health has been under developed.

In a policy environment, the production of small fragments of abstracted evidence is vulnerable to misinterpretation. An example is the finding that the average mental health decrease associated with housing becoming unaffordable is around two percentage points on a 1–100 scale [10]. Though this example infers that the total measurable housing effect on health is two percentage points, the evidence is limited in referring to (quite strictly defined) unaffordable housing and ignores all other housing bundle components such as location, condition, suitability. In attending to the limitations of a methodological framework based on abstraction, this paper proposes a more holistic conceptualisation of housing and the broader social conditions that may influence health and wellbeing. We propose that housing bundles may be a more appropriate unit of analysis as they allow us to capture the influence of housing across and beyond affordability, location, security, and even amenity. Others have previously recognised the compound relationships between housing and wellbeing; important among these is Saegert and Evans [21] who note the “cascade of troubles” that befall many disadvantaged households.

In this paper, we propose and describe an index to capture the means by which housing bundles influence health and wellbeing. Borrowing from a body of work in public health [22], which defines health risks to the individual as potential “insults”, we conceptualise the measure as reflecting combined housing “insults to health”—an Index of Housing Insults (IHI). We then apply this index to a sample of just over 1000 low-income households in Australia. We test relative exposure and estimate the scale and pattern of associated health effects. To test the effectiveness of the index over and above the more parsimonious measures, such as tenure, we undertake multivariate regression modelling to account for confounding.

The analysis is structured around two research questions:

(1) Who in our population is more exposed to an accumulation of housing insults?
(2) Is there a corresponding gradient across a range of health measures (mental health, physical health, general health and clinical depression)?

### 1.2. A Bundle of Housing Insults?

There is a substantial and growing evidence base examining housing factors and their direct and indirect effects on health outcomes [23]. Characteristics of the overall quality [24] and condition of dwellings (for example, damp [13], warmth [25] and thermal quality [26]) are now well established as important influences on health outcomes. In both separate quantitative analyses and systematic reviews (for example [25,27]), housing quality and condition has been shown to affect health outcomes, such as respiratory illness [12,14], mental and physical health [13,28], or cardiovascular disease [29].

Tenure has been shown across a large number of studies to be directly or indirectly related to health and wellbeing outcomes (for example [30]), and looking in the other direction, Smith [31] drew attention to the role of health status as a determinant of housing tenure opportunities. In the majority of studies, people who rent have lower health on average than homeowners and home purchasers. Much of the explanation of this difference is attributed to characteristics of the different tenures, for example higher levels of ontological security provided by homeownership [32,33]. Some caution, however, is required when considering the effects of tenure on health and wellbeing, because alongside robust evidence of the effects of tenure in some contexts and studies (for example [34]), home ownership is associated with income and wealth accumulation in most industrial and post-industrial economies. Using the example of Australia, homeownership is a proxy for income status, and lower-income households are more likely to rent [35]. This means that some explanation for the poorer overall health among renters can be attributed to who rents, rather than the tenure itself [36]. The recent study by Mason et al. [37] highlighted the additional complexity of the influence of tenure over individual outcomes, finding evidence of an interaction between tenure and affordability which resulted in renters being more vulnerable than home owners to the health-related effects of housing affordability. In addition to tenure security, there is a small but convincing literature linking tenure and housing security [38] or fear of crime [39] to individual health and wellbeing outcomes. These effects tend to be focussed upon mental rather than physical health (for example [40]).

There is a substantial body of recent work that aims to isolate the health and wellbeing effects of housing affordability. Not only are affordability problems associated with poorer overall health and specific health problems such as arthritis [41] or depression [42], housing affordability has also been shown to affect health directly (for example via foreclosure or possession [43,44], mortgage arrears [11], rental insecurity [37] or fuel poverty [45].

Perhaps the most substantial body of literature in the field establishes the health and wellbeing effects of the condition and quality of dwellings. A systematic review of the health effects of housing improvement undertaken in 2001 [46] found health gains from housing-based interventions. In their review, Krieger and Higgins [47] describe existing evidence of housing quality effects across a broad range of health and wellbeing outcomes. Housing conditions may dictate exposure to smoke, chemicals or toxins, [48] and also directly influence mental health or inhibit adequate social interaction [49,50]. Most recently, results from the GoWell study in Glasgow find positive associations between improvements to dwelling fabric and mental and physical health [51]. In assessing the evidence across much of this work, the recent Cochrane review [52] examined studies of health change attributed to housing improvement. This extensive review highlighted the importance of housing interventions that improved warmth and provided adequate space as most clearly related to health improvements of residents.

Finally, there is a convincing wave of studies linking features and quality of the neighbourhood environment with measured health and wellbeing outcomes. Among the recent studies of interest, Jones-Rounds et al. [53] find that not only does poor neighbourhood quality (in this case, a composite measure of satisfaction across areas such as with neighbourhood environment, accessibility, safety and disorder) contribute to lower psychological wellbeing, but that satisfaction may also “buffer” some of the negative effects of housing conditions.

## 2. Materials and Methods 

To explore our research questions, we examine the combined impact of multiple components of housing on health and wellbeing. This analysis is based on data from a postal and online survey administered in 2013 to 1008 low-to-moderate income South Australian households, including a booster sample of households in poor condition dwellings in disadvantaged local areas—the Health and Wellbeing Survey. Completed by a named tenant, mortgage holder or owner in each household, the survey was designed to provide measures suitable for inclusion in the IHI and builds upon survey instruments previously used elsewhere [54]. The sample intentionally focussed on the population most often implicated in health effects of poor housing—lower income, recipients of assistance and living in poorer quality dwellings. It comprised approximately 100 questions, including standardised measures of physical health, general health, mental health and diagnosed depression; questions about employment, perceptions of the impact of housing assistance and demographic, economic and locational accessibility. The survey was specifically designed to test the relationship between health and housing, and we are unaware of another dataset of similar scale and focus in Australia. Building on an established housing literature, the survey also collected detailed information about factors such as residential security, quality, affordability and satisfaction that have been shown to be critical for the residential bundles that individuals and their households assemble (for example [55,56,57,58]). Housing components implicated in possible health outcomes were selected and are detailed in Table 1 below.

The index aimed to capture the multiple small insults that housing may make upon the lives of individuals. The inclusion of multiple housing insults stands in contrast to analyses that restrict consideration of the relationship between housing and health to a single measure, such as dwelling quality or tenure. Evans, Li and Whipple [59] discuss the advantages of formulating multiple risk factor exposure into a composite score. We note that developing a meaningful index is not straightforward and there is no specific procedure to follow [60]. As there is little precedent for the construction of the IHI, we used an inductive approach informed, in part, by our previous use of indices (for example [61]) to assess complex social and economic phenomena.

For the first-stage of the analysis, we combined the unweighted component indicators described in Table 1 (noting that the term is used in the sense that no empirical method is used to derive weights and so the individual data items have equal weight to the extent that each has a range of zero to one) [62], each normalised to have a range of zero to one [0,1]. After individual components were normalised, a linear additive index was constructed, where each indicator was given equal weight. We note alternative methods available to construct formative indicators (i.e., indicators are viewed as causing an individual’s level or rank in the scale representing the severity of housing insults—in contrast to a reflective indicator model in which observed indicators are responding to the underlying factor). The aggregate index was then normalised to a range of zero to one hundred [0,100] (x_new_ = (x − min(x))/(max(x) − min(x))).

The formative model is therefore a linear index of the form:

(1) $$\eta =w_{1}x_{2}+w_{2}x_{2}+\ldots +w_{p}x_{p}$$

Since model weights cannot be estimated for this formative model, and there is no theoretical guidance as to appropriate weights for combining the indicators, either equal (*w*_1_ = *w*_2_ = *w*_3_ … = … *w*) or unequal subjective weights are imposed or weights are derived by an empirical method such as Principal Component Analysis (PCA). By design, PCA aims to be a summary of related input variables, therefore it will be less successful if the indicators are not related. This is a consideration for the construction of the IHI. Because the index captures the trade-off and bundling of housing attributes: it *therefore includes indicator variables that are not highly correlated*—a bundle of similar attributes will not necessarily be highly correlated with an alternative trade-off bundle of attributes. This is important conceptually—because these variables are not highly correlated, no individual variable can serve as a surrogate for, or indicator of, the other variables. Therefore, the indicator variables are selected to represent trade-offs and bundles and consequently are, by design, not necessarily appropriate for PCA. Nonetheless, to confirm that the trade-off-bundle frontier acts as expected, PCA was conducted as an exploratory analysis—noting that if indicators are not correlated at about 0.3 there is no expectation that PCA will be successful (there is little evidence of a linear relationship between indictors). As shown in Table 2, correlations between the set of indicators varied—ranging from −0.08 to 0.79.

Using (a single-component) PCA, based on polychoric correlations of the ordinal data, to derive weights to construct a linear index results in an indicator that has a correlation coefficient of 0.919 with the simple unweighted construct. It is an interesting artefact of the data that, notwithstanding relatively low correlations between some pairs of variables, the PCA-based predicted aggregation is highly correlated with the simple unweighted index. For the purposes of this initial exploration, the unweighted index is therefore applied, though we flag a detailed testing and sensitivity analysis as part of the later development of this index.

Missing data is a common problem in survey data, but for these indictors the maximum missing data has only two absent observations out of 640 (<0.4 per cent) missing, which for survey data questions is at least acceptable. For construction of the index, the summation process excludes missing data at the individual variable level.

In interpreting the index, an increase in the index corresponds to worse housing as measured across the indicators summarised above. Correspondingly, a lower index value represents exposure to fewer housing insults.

It is important to note the low degree of correlation between component indicators (as shown in Table 2). The correlation between most items was low, for example ‘affordability’ and ‘tenure security’, or ‘access to services’ and ‘house condition’, and in some cases zero or negative correlation (for example ‘tenure security’ and ‘feeling safe in the neighbourhood’). This suggests that the components of the index can be, to a large extent, regarded as capturing distinct parts of a housing bundle. There are a small number of index components in the matrix that have a relatively high correlation (for example ‘dwelling adequacy’ and ‘dwelling condition’). This suggests that these two components overlap to some extent in their effect on the housing bundle. If component indicators were found to have high correlation values across several other components, they would have been excluded from the analysis. This was not the case, and therefore all indicators were retained. We additionally conducted Variance Inflation Factor (VIF) sensitivity tests and these suggested no multicollinearity in either the index components or the explanatory variables (results not shown). A sensitivity test for outliers (using the BACON algorithm [63]) concluded that there were no outliers. To explore the pattern of association between the IHI and health outcomes, we examined four health measures. Two came from the SF-12 (mental and physical health which are calculated to have a possible (population) range of 1–100). The third is a self-rated health ordinal scale with five options ranging from excellent to poor, and the fourth is diagnosed clinical depression and is a binary yes/no variable.

To analyse mental and physical health (measured on a 0–100 scale), we use the standard linear regression model (an Ordinary Least Squares (OLS) estimator). Coefficients for the OLS are interpreted either as the marginal change in the dependent variable (DV) for a small change in the explanatory variable or, for dummy explanatory variables, the difference in the DV between the two states the explanatory dummy can take. For general health (measured on a 5-point scale from excellent to poor), we use a non-linear ordered logit model and for diagnosed clinical depression (a binary measure, yes/no), the standard non-linear logit model. For the ordered logit or the logit models, the coefficients are converted to odds ratios (OR). An OR of 1.0 indicates no effect; greater than one indicates that the variable increases the odds associated with the DV; odds ratio less than 1.0 indicates that the variable decreases the odds.

## 3. Results

### 3.1. Descriptive

Descriptive sample characteristics are presented, alongside corresponding mean sample IHI scores in Table 3. Across each of the characteristics, there is a strong apparent association. The table shows a clear age patterning, where increasing age is related to exposure to lower housing insult in a strongly linear pattern. This is most clearly demonstrated by comparing the mean IHI of the youngest group (17–24 years: 45) with that of the oldest group (65 years and older: 33). We temper these results with an acknowledgement of an established, positive association between age and residential satisfaction (for example [64]), but interpret the age gradient of the IHI to be over and above this. Historically, the majority of Australians have avoided poverty in older age through their housing, especially outright owner occupation [65], although recent changes in the role of housing over the life course [66] have placed this relationship at risk.

Similarly, there are substantial differences in the mean IHI scores across labour force status. The unemployed have the highest mean IHI, followed by those not in the labour force and those in part-time employment. The full-time employed have a very low mean IHI score (29). Considering marital status, those classified as married/de facto have the lowest mean IHI score (32), followed by widowed, separated, divorced, and the cohort of ‘never married’ has the highest mean IHI score (41). There was no substantial gender difference in mean IHI scores. 

The participants with a long-term disability or health condition had an average IHI (39), which was above the overall mean (36) and the average IHI score for people with no long-term disability or health condition. Among people living in a household where someone else had a disability or long-term health condition, the mean IHI was also 39. There is a strong, linear gradient in the IHI from those with excellent self-assessed health (25) to those with poor health (43). The average difference in IHI score for those with and without diagnosed clinical depression was also marked. Those respondents with clinical depression had a substantially higher mean IHI.

The participants identifying as Indigenous Australians had a mean IHI of 35, which is a slightly lower mean IHI than among the non-Indigenous population (36). This finding (acknowledging the relatively small number of Indigenous respondents in the sample) and the concentration of Indigenous housing disadvantage in remote areas where the survey was unlikely to reach may reflect a local policy focus on providing adequate housing to Indigenous persons. This is a topic worthy of further investigation in subsequent analyses.

Finally, there was a substantial difference in mean IHI scores across housing tenure types, where home owners had the lowest mean IHI (29), compared to social housing tenants (36), and private renters receiving no assistance who had a very high mean score (49).

### 3.2. Bivariate Analysis

**Figure 1:**
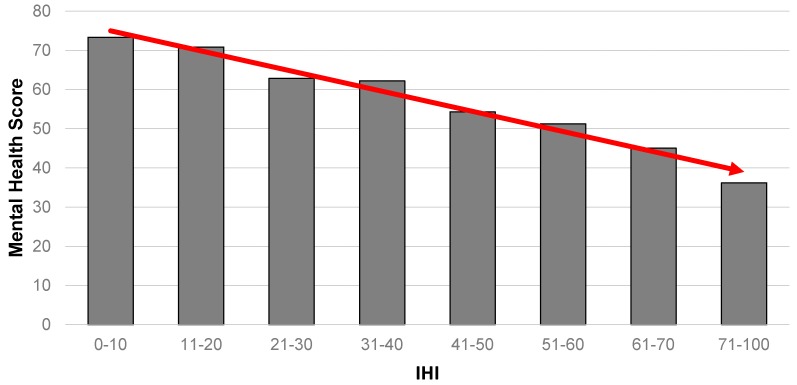
Association between mental health (from SF-12) and IHI (*n* = 615). Note: altered scale (71–100) to account for relatively small numbers at the extreme end of the IHI continuum.

In order to explore the relationship between the level of housing insult and health, we examine four health outcome measures—mental and physical (from the SF-12), self-reported general health and diagnosed clinical depression. Figure 1 summarises the results for the relationship between mental health and the IHI. It portrays a strong gradient in the data, where those with the highest level of housing insults are shown to be highly likely to have the poorest mental health.

**Figure 2:**
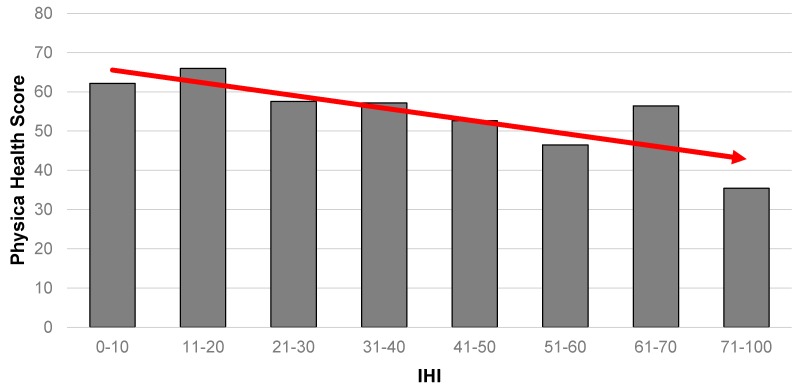
Association between physical health (from SF-12) and IHI (*n* = 615). Note: altered scale (71–100) to account for relatively small numbers at the extreme end of the IHI continuum.

A comparable result is also evident for physical health (Figure 2), where those with the worst level of physical health were also the most likely to have the highest IHI scores. Notably, the gradient is less obvious than for mental health.

**Figure 3:**
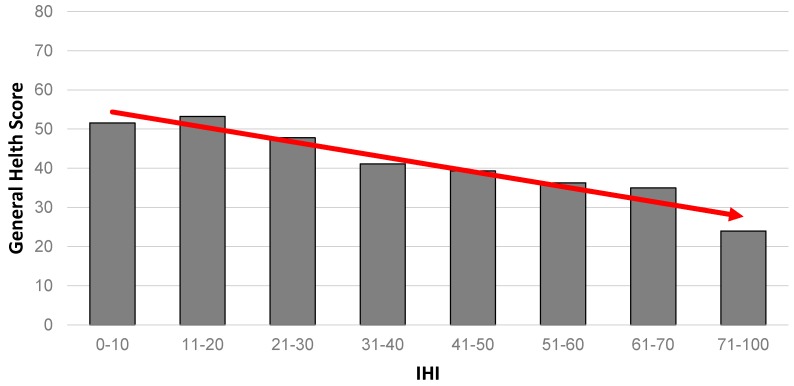
Association between self-rated health and IHI (*n* = 634). Note: altered scale (71–100) to account for relatively small numbers at the extreme end of the IHI continuum.

An almost identical gradient pattern seen for mental health is repeated when we consider self-rated general health as an outcome (Figure 3).

**Figure 4:**
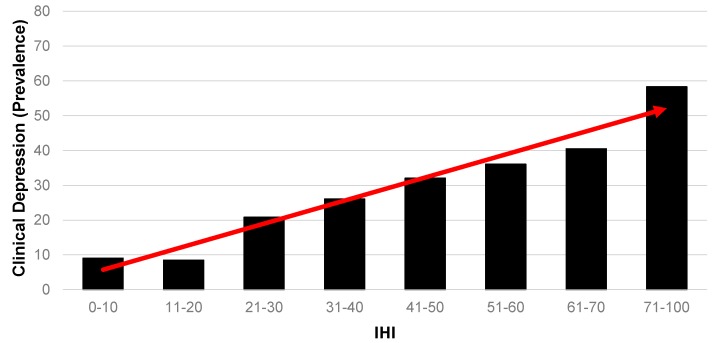
Association between (self-reported) prevalence of diagnosed clinical depression and IHI (*n* = 638). Note: altered scale (71–100) to account for relatively small numbers at the extreme end of the IHI continuum.

Figure 4 presents a different outcome. Survey respondents were asked to self-report if they had been diagnosed with a number of health conditions, one of which was clinical depression. Across the 638 respondents for whom an IHI could be constructed in this sample, a substantial 26 per cent had been diagnosed with depression. Because this is such a dominant diagnosed health problem among this population, it is presented here in terms of relative likelihood. In this case, the gradient should be interpreted in the opposite direction to Figure 1, Figure 2 and Figure 3. The figure clearly shows a strong gradient demonstrating a high likelihood of clinical depression with a greater exposure to housing insults.

Overall, this analysis of association between level of exposure to housing insults and the corresponding health and wellbeing characteristics of individuals shows a relationship of substantial policy significance. People with poor health also have the highest exposure to housing problems—that are likely to affect their health further, flagging the existence of a substantial mismatch between exposure to potentially harmful housing and the individuals within a population that arguably have the greatest need for housing to protect or improve their health. The results are, however, tempered by the need to acknowledge unexplained confounding (i.e., factors that explain poor housing may also explain poor health), reverse causation and selection bias (for example, people with existing health problems are necessarily more vulnerable within the housing market because they often have low or statuary incomes and are therefore more likely to be forced to trade-off elements of housing quality for affordability and access).

### 3.3. Multivariate Analysis of Outcomes

Building on the evidence provided by the index of a strongly graded relationship between exposure to housing insults and corresponding exposure to health problems, we undertook a second series of analyses utilising multivariate regression. This second analysis allowed us to account for confounding and compositional bias as well as test the degree to which the IHI captures vulnerability to housing problems compared to other more simple proxy measures, such as income or tenure.

The results of the analysis are summarised in Table 4 and Table 5. Table 4 shows that on average, over and above people’s gender, tenure, education, age, labour force, marital status, income, disability and carer characteristics, there is a strong and highly significant relationship between level of exposure to housing insults (IHI) and mental health, but no significant relationship for physical health (dummy variable results are detailed in Appendix A, Table A1). Thus, for mental health, the value of the coefficient for the IHI (−0.246, *p*-value = 0.000) can be interpreted as, all other explanatory variables held constant, the amount of change in mental health associated with one point change in the IHI. Noting that both the IHI and the health outcome measures are on a 100-point scale, this means that for every 1-point increase in IHI, there will be a corresponding 0.246 decrease in mental health. In practical terms, this means that relatively small differences in accumulated exposure to housing problems are implicated in sizeable differences in mental health. For physical health, however, we find no evidence of statistical significance at commonly stated levels of acceptance (*p*-value 0.301). To some extent, this is unsurprising and fits with previous findings of limited or significantly lagged physical health effects of housing limitations—important exceptions within the literature are injury due to dangers in the dwelling [67] or housing problems related to extreme climates (for example [68]).

For the non-linear models for self-rated general health and clinical depression, all other explanatory variables held constant, we find strong evidence of statistical association (in both models the *p*-value on the IHI is 0.000). For general health, the odds ratio for the IHI is 0.965, indicating that a one unit increase in the IHI is associated with a decrease in the odds of having higher general health of about 3.5 per cent (100–96.5). On the other hand, an increase in the IHI of one unit is associated with an increase in the odds of clinical depression by approximately 3.2 percent.

Overall, we find that, after controlling for an extensive number of possible confounders (^#^), the IHI provides additional predictive power to the explanation of levels of mental health, general health and clinical depression beyond the more traditional measures such as tenure or income. For physical health, however, the IHI provides no additional predictive power.

## 4. Discussion

The analysis described above has been guided by two research questions:

### 4.1. Are Some Groups within Our Population More Exposed to Combined Housing Insults? 

The descriptive table (Table 3) shows population cohorts within the sample that appear vulnerable to an above average level of housing insult. This table highlights the vulnerability of key groups within the (already low-income) sample. Unsurprisingly, younger people, unemployed people and single people are shown to have the most health-adverse housing in the sample. The other dominant association evident in the table is between existing health disadvantage and high level of housing problems. Those households containing individuals with poor health or disability appear to be especially vulnerable. Notably, people with clinical depression were living in some of the most health adverse housing conditions as measured by the IHI (44 compared to the population average of 36).

Disentangling the relationship between housing and health to determine whether poor quality housing results in poorer health or whether lower-income persons with poorer health are simply forced by market processes into ‘health-risky’ dwellings will need to await later analysis. However, we can note the strong association between poor health, poor housing and vulnerability within the labour market. Recent policy shifts in many nations (such as England, Australia [36], The Netherlands [69], Scotland [70]) to reduce government investment in social housing may exacerbate these problems, adding to the burden of acute health care and further entrenching inequality for the most disadvantaged. Across each of these jurisdictions, social housing declined as a proportion of the national housing stock in the first decade of the 21st century [71] and, looking to Australia, there has been a well-documented and long-run tenure shift towards the private rental tenure [72,73] and a corresponding shrinkage of the social rental sector and the proportion of outright home owners.

### 4.2. Is There a Corresponding Health Gradient (Across Mental Health, Physical Health, Self-Rated General Health and Clinical Depression Outcome Measures)?

Across all the health outcomes examined and prior to adjustment for confounding by socio-demographic factors, there was an obvious gradient. A greater level of housing insults corresponded with worse physical, mental and self-assessed health as well as the pronounced prevalence of diagnosed clinical depression. These outcomes provide strong evidence of a relationship between health-adverse housing and poorer health in this group of lower-income Australians. Gradients such as these are observed for a number of economic factors, including income and employment status vis-à-vis health and have been discussed in both academic and policy documents, such as the World Health Organisation (WHO) Report on the Social Determinants of Health [74]. These gradients, however, are less often measured and discussed in relation to housing and health. The generation and application of the IHI in this study has allowed us to generate compelling evidence for action across a spectrum of households in Australia to ‘flatten’ or equalise these observed gradients.

The second series of analysis utilising multivariate regression provides additional evidence that (in the case of mental health, general health and depression) the association with IHI is strong and highly significant, a relationship that holds even after we account for the influence of an extended range of socio-demographic characteristics. The lack of significance in the physical health model indicates that the gradient observed in the simpler bivariate analysis is likely to be largely explained by non-housing influences. The scale of effect for mental health, general health and depression is an important finding, suggesting that even small improvements in housing conditions may have large potential effects on health for this group.

It should be noted that even after adjustment for confounding in multivariate models, we cannot comment on the direction of effects underpinning the associations observed in these analyses. Our findings should be interpreted in two ways—that those with the worst health are highly likely to be in poor housing and that those with poor housing are likely to have the worst health.

## 5. Conclusions

This paper has proposed an encompassing means to examine and document the housing and health relationship, one that acknowledges the combined influence of housing bundles. We test this index on a large sample of lower-income Australians, but highlight the potential value of such an approach in broader population analyses. In order to capture the combined influence of multiple housing problems on health, we constructed and then examined an Index of Housing Insults among a lower-income population in Australia. Promisingly, this exploration both confirms and questions our thinking about the mechanisms by which housing acts in individual lives. It also gives clear direction for new, more causal work.

In conceptualising the index, we capture a bundle of multiple components that may be working together. Our analysis indicates that this is a potentially valuable approach, and it suggests that distinct housing components may act in concert upon individuals. There is evidence of overlap between the components of the index that we have tested in this paper, nevertheless, the analysis indicates that multiple housing insults might act simultaneously and demonstrate a larger deleterious effect on health. This simple finding may be used to question the appropriateness of proxy indicators for assessing the influence of housing on individual outcomes (such as mental health)—we have, for example, used housing affordability in this way previously.

Because housing (and importantly its deficit) is so closely tied to income and wealth in post-industrial countries, findings such as those presented in the paper beg the question—does the analysis simply measure poverty? We suggest that although housing is a commodity where components of dwelling suitability, location, safety and quality are embedded within the price, there are other important, competing (and sometimes dominating) influences (across and beyond health and housing career) [75]. Moreover, at the level of both the individual and identifiable populations, health outcomes cannot simply be ‘read off’ against income. We observe in this current analysis evidence of a bundle of housing insults that operates over and above pre-existing health and (lack of) income to affect health. For many lower-income people, housing bundles act alongside and in addition to broader poverty, exposing them to the double disadvantage from both poverty and accumulated housing problems.

This study has a number of potential limitations that should be acknowledged. It is based on a sample of just over 1000 lower-income dwellings where external housing conditions were poor or previous housing assistance had been received. Though the sample selection prevents us from making conclusions that apply to the whole population, we can of course make more focussed statements about the effect of housing problems on the population known to be especially vulnerable to the health effects of housing. The findings should also be considered with awareness of potential bias from missing data. The analytical dataset is limited to respondents for whom complete data was available. The extent to which the findings of this study on the associations between housing and health in similar populations can be generalised internationally should be guided by an acknowledgment of the relatively good overall condition of the Australian dwelling stock, relative housing affordability and the size and allocation policies of the social and private rental sectors. The sample size is adequate to detect associations between the IHI and several measures of health and wellbeing, but we note the value of any future, more extensive data collection. This suggests a need for future work to consider in-home exposure to housing insults. It is well known that some demographic characteristics (for example, children or older people) predispose individuals to more time in the home, and this may affect the action of housing insults on the individual. Intrinsic to the design of this study, the housing components of the index are self-assessed. We note that this may be correlated with the health self-assessments also measured, such that people with poorer mental health may be more likely to rate their housing conditions as poor. We acknowledge this as a limitation and suggest that further developments of the index could include more objective housing quality measures, such as those discussed in [76].

An additional limitation of this study relates to its cross-sectional nature. We acknowledge the inability of point in time analyses, such as this one, to reveal causes and consequences of the relationship between housing and health or acknowledge the effects of differential exposure. To a large extent, we present this analysis as a contribution to conceptual thinking that can eventually applied longitudinally. To date, detailed housing quality data has been limited in Australia—the last national housing conditions survey [77] was undertaken in 1999—but there is a developing focus for this data in funding and research and a call for any new data to be longitudinal.

Overall, this analysis finds value in the ‘bundle’ approach to examining housing problems. By shifting our focus from measuring the effect of separate housing components, to measuring the effects of people’s whole bundle of housing, we are likely to produce larger effect sizes and more convincing (and arguably more accurate) policy arguments. Questions remain for policy makers, however, around how to use this evidence and what the policy focus should be? Can interventions be bundle-focussed? Is there a means of prioritising bundle components?

This paper began with a reference to John Snow, the medical practitioner who cut short a typhoid epidemic by intervening in the built environment to produce better population health. While Snow intervened to achieve a single outcome, social reformists and advocates who followed him worked to establish better housing standards across a number of dimensions of health. Such efforts contributed to improvements in the public health aspects of housing stocks, the introduction of minimum dwelling standards in many developed nations, the establishment of town planning as both an academic discipline and a profession, and the eventual foundation of large-scale public housing in many nations. Over recent decades, the appetite for such policy interventions has waned with the rise of neoliberal philosophies of government [78], the emergence of a society that accepts a degree of human-created ‘risk’ [79] and a resultant requirement for governments to increasingly justify the external benefits of subsidising good housing [80]. The analysis presented in this paper flags the risk that changing housing regimes in developed nations may have negative impacts on health across a number of domains. Many of these impacts may be small and difficult to observe, but their combined consequences for affected individuals will be substantial. The cost of such adverse health impacts will be borne by individuals in terms of their quality of life, capacity to find paid work et cetera and in terms of their health as well as by society in terms of public sector outlays on acute health. Researchers and societies need to embrace a broader understanding of the relationship between housing and health in order to inform the community and their governments of how best to improve housing and public health.

## Figures and Tables

**Table 1:** Index components.

Affordability Domain
Is housing affordable? Number of problems because of shortage of money over the last 12 months?
Security Domain
3. Is housing tenure secure? 4. Do you feel safe in your residential neighbourhood?
Quality of Dwelling Domain
5. How well does your dwelling meet the needs of you and your family? 6. Count of identified dwelling problems 7. Does dwelling meet personal care needs? 8. What is the state of repair of your dwelling?
Quality of Residential Area Domain
9. Count of identified problems in the local area
Access to Services and Support Domain
10. Does dwelling meet needs for access to services? 11. Does dwelling meet needs for family support?

**Table 2:** Matrix of index component correlations.

		1	2	3	4	5	6	7	8	9	10	11
1	Housing affordability	1										
2	Financial problems	0.09	1									
3	Tenure security	0.12	0.13	1								
4	Residential safety	−0	0.32	0.00	1							
5	Dwelling adequacy	−0.1	0.38	0.31	0.50	1						
6	Physical dwelling problems	−0.1	0.49	0.20	0.44	0.66	1					
7	Dwelling meets personal care needs	0.17	0.04	−0.1	0.12	0.17	0.07	1				
8	Dwelling condition	0.02	0.35	0.22	0.38	0.68	0.59	0.14	1			
9	Neighbourhood quality	0.00	0.10	−0	0.15	0.09	0.10	0.01	0.08	1		
10	Dwelling access to services	0.15	0.15	0.02	0.19	0.23	0.15	0.44	0.11	0.03	1	
11	Dwelling access to family support	0.25	0.04	0.11	0.13	0.17	0.08	0.79	0.15	0.02	0.60	1

**Table 3:** Summary table of mean index of housing insults (IHI) by selected socio-demographic characteristics (*n* = 638).

Index of Housing Insults	Mean	*n*	95% CI	
Population age	36	638	35	38
**Age cohort**				
17–24	45	12	36	54
25–34	35	33	29	42
35–44	39	117	36	43
45–54	36	134	33	39
55–64	36	130	33	39
65+	33	200	31	35
**Long term disability/health condition**				
Yes	39	309	37	41
No	34	329	32	35
**Labour force/employment status**				
Full-time employed	29	91	26	32
Part-time employed	35	103	31	38
Unemployed	42	32	36	48
Not in Labour Force (NLF)	37	396	36	39
**Self-rated general health**				
Poor	43	72	39	47
Fair	40	192	38	43
Good	35	236	33	37
Very good	30	109	27	33
Excellent	25	25	20	29
**Marital status**				
Married/de facto	32	233	30	35
Widowed	33	78	30	37
Divorced	39	161	36	41
Separated	38	43	33	43
Never married	41	118	37	44
**Tenure**				
Home owner/purchaser	29	234	28	31
Private rent (no assistance)	41	45	37	45
Rent assistance	49	103	46	52
Public renter	36	252	34	38
**Gender**				
Male	35	233	33	38
Female	37	401	35	38
**Indigenous**				
Yes	34	23	28	41
No	36	590	35	38
**Carer**				
Yes	37	166	35	40
No	36	439	34	37
In your household	39	130	36	42
Elsewhere	35	52	30	40
**Clinical depression**				
No	33	474	32	35
Yes	44	164	41	47

**Table 4:** Linear regression models for continuous mental and physical health measures (*n* = 471) ^#^.

	Mental Health	Physical Health
IHI	−0.2457 ***	−0.0314

^#^ Adjusted for gender, tenure, education level, age, labour force status, marital status, income, disability and carer status. *** Highly statistically significant <0.0001.

**Table 5:** Non-linear regression model odds ratios (ORs) for general health (*n* = 484) and diagnosed clinical depression (*n* = 462) ^#^.

	General Health OR	Diagnosed Clinical Depression OR
IHI	0.9651 ***	1.0323 ***

^#^ Adjusted for gender, tenure, education level, age, labour force status, marital status, income, disability and carer status. *** Highly statistically significant < 0.0001.

## Appendix A

**Table A1:** Dummy control variable results for linear regression models.

	Mental	Physical
Dummy Control Variables		
Gender *(ref. male)*	1.6224	−1.0788
*Tenure (ref. home purchaser)*		
Own House	3.5182 *	0.7391
Private Renter	4.6015 ***	−0.9704
Public Renter	−1.3851	−1.5037
Other	2.8258	1.3821
*Education (ref. did not complete high school)*		
SACE/High school	−0.4048	−1.2277
Trade/Apprenticeship	2.4696	−1.5958
Certificate	−1.6421	1.471
University	−3.0851	1.5361
*Age group (ref. >76 years)*		
17–25	−7.8523	8.8588 **
26–45	−4.9865	7.3293 ***
46–55	−4.2839	6.9478 **
56–65	−1.1965	1.4824
66–75	−3.1916	2.0587
*Labour force (ref. employed full-time)*		
Part-time/Casual	−3.7535 *	−0.6195
Unemployed	−4.4467	−1.5896
Home	−5.9406 **	0.3693
Retired	−0.2594	−3.987
Student	−9.0457 **	−0.1002
Unable	−5.1836 **	−6.0085 ***
*Marital status (ref. married/partnered)*		
Widowed	−3.1065	−1.0714
Divorced	−0.5377	−1.6026
Separated	−0.1712	−0.8565
Never Married	0.4993	0.1727
*Weekly Income (ref. A$250–$499)*		
No Income	2.6923	1.0862
$1–249	−1.7453	−0.3451
$500–799	0.5104	0.852
$800–1199	−0.8757	0.1218
$1200–1699	−2.6891	0.1918
$1700–2499	1.3031	−0.2972
$2500–3499	−1.2598	−2.1897
$3500 plus	−8.8644	11.1137
*Carer (not a carer)*	−0.614	−4.6530 ***
*Disability (ref. no disability)*	−3.7520 ***	−9.3320 ***
_cons	60.7331 ***	45.8851 ***
N	471	471

***, **, * Statistically significant at 1%, 5% and 10% levels respectively.
